# Practical examination of bystanders performing Basic Life Support in Germany: a prospective manikin study

**DOI:** 10.1186/1471-227X-8-14

**Published:** 2008-11-20

**Authors:** Christoph HR Wiese, Henryk Wilke, Jan Bahr, Bernhard M Graf

**Affiliations:** 1 Department of Anaesthesiology, Emergency and Intensive Care Medicine, University Medical Centre Göttingen, Germany

## Abstract

**Background:**

In an out-of-hospital emergency situation bystander intervention is essential for a sufficient functioning of the chain of rescue. The basic measures of cardiopulmonary resuscitation (Basic Life Support – BLS) by lay people are therefore definitely part of an effective emergency service of a patient needing resuscitation. Relevant knowledge is provided to the public by various course conceptions. The learning success concerning a one day first aid course ("LSM" course in Germany) has not been much investigated in the past. We investigated to what extent lay people could perform BLS correctly in a standardised manikin scenario. An aim of this study was to show how course repetitions affected success in performing BLS.

**Methods:**

The "LSM course" was carried out in a standardised manner. We tested prospectively 100 participants in two groups (**Group 1: **Participants with previous attendance of a BLS course; **Group 2: **Participants with no previous attendance of a BLS course) in their practical abilities in BLS after the course. Success parameter was the correct performance of BLS in accordance with the current ERC guidelines.

**Results:**

Twenty-two (22%) of the 100 investigated participants obtained satisfactory results in the practical performance of BLS. Participants with repeated participation in BLS obtained significantly better results (**Group 1: **32.7% vs. **Group 2: **10.4%; p < 0.01) than course participants with no relevant previous knowledge.

**Conclusion:**

Only 22% of the investigated participants at the end of a "LSM course" were able to perform BLS satisfactorily according to the ERC guidelines. Participants who had previously attended comparable courses obtained significantly better results in the practical test. Through regular repetitions it seems to be possible to achieve, at least on the manikin, an improvement of the results in bystander resuscitation and, consequently, a better patient outcome. To validate this hypothesis further investigations are recommended by specialised societies.

## Background

Basic Life Support (BLS) by lay people has a special importance in the care of emergency patients. First aid and BLS are in the first three links of the chain of rescue [1,2]. Even the internationally recognised "chain of survival" includes two, in the presence of an automated defibrillator even three, of four links that have to be performed by lay people [3]. Therefore, lay help during cardiac arrest is one of the requirements for a successful resuscitation of a patient in terms of a return of spontaneous circulation (ROSC) [4-9]. However, lay people can only play their role within the chain of survival if they are adequately trained and if continuous repetitions of relevant training contents are offered and used [8,9].

Strengthening of BLS and shortening of the therapy-free interval in emergency situations are important goals of the existing guidelines especially during resuscitation [3,8-12]. In the current guidelines of the European Resuscitation Council [3] a simplification of BLS was recommended to reach the earliest possible start of resuscitation. Therefore the special value and importance of bystander resuscitation has been clearly confirmed in numerous studies [[2,3,5,7], and [8]].

Besides it is important to increase the helpfulness of lay people in emergency situations. Previous surveys showed that, due to various reasons, lay people in only 50% of the medical emergency situations they were confronted with, started to provide the necessary assistance [13,14].

There are lots of factors influencing the behaviour of lay people in emergency situations. The feeling of competence appeared to be an essential factor for the behaviour of bystanders. The feeling of competence highly depends on the presence of theoretical and practical knowledge, which in turn must be imparted by respective courses [4,15]. High quality of BLS training is especially important for the motivation of lay people so that as a consequence competence is increased and thus adequate help can be provided [[8,9], and [16]].

Considering the great importance of first aid, in the current study we investigated to what extent lay people could perform BLS correctly in a standardised manikin scenario. The test followed after the respective one day first aid (LSM) course. It was also investigated how course repetitions affected success in performing BLS. The test referred to the teaching contents of the course on BLS in accordance with the ERC guidelines dating from 2005 [3].

However, in Germany most people attend a course on emergency measures only once in their life, as at the moment there is not any legally binding repetition of such courses. Nonetheless specialised societies (such as European Resuscitation Council) try to optimise the emergency training of the population as far as possible through new algorithms and training offers [3].

The main aim of the investigation was the comparison of the performance of BLS between the two defined groups (**Group 1: **Participants with previous attendance of a BLS course; **Group 2: **Participants with no previous attendance of a BLS course). Therefore we want to show the practical examination of BLS bystanders concerning their previous attendance of BLS courses.

## Methods

In a period of three months all 100 participants of "LSM courses" were included in the present investigation. An average of ten participants attended each course. The consecutive sampling technique minimizes selection bias with the caveat that the study's results are applicable to those individuals who self-select to sign up for the "LSM course". This group was representative to the overall population.

The duration, the contents and the teaching materials followed the guidelines for conducting "LSM courses" in Germany. These details, including trainers' qualification, are uniformly predefined in accordance with the German Licensing Regulation, so that the courses are comparable nationwide concerning contents and time. The scheduled time for BLS amounted to 120 minutes (theory and practice). All the courses were carried out by the same instructor, guaranteeing low intraobserver bias. The course instructor held a relevant training qualification valid in Germany as a so called "First Aid instructor" and as "LSM course instructor". The participants were tested by a second person who was not involved into the course concept. No specific instructions were given to the participants. All tests were carried out by the same person. Therefore the testing of the participants was blinded. Knowledge and skills of the participants were not tested before the educational intervention.

Concerning their previous BLS-knowledge (previous attendance of a "LSM course"), the participants were divided into two groups (**Group 1: **Participants with previous attendance of a BLS course; **Group 2: **Participants with no previous attendance of a BLS course). During the standardised course both groups were mixed. Participants were divided into the groups after the educational intervention.

The data and results were prospectively compiled through the recording software of the resuscitation simulator (Laerdal Advanced Resuscitation Anne Skill Trainer™, Laerdal Skill Software™: e.g. effectiveness of breathing, chest compressions, and whole scenario time) as well as through video recording (DVD recorder: e.g. shout for help and check for breathing) and were evaluated retrospectively. The NFT was recorded using the same stopwatch and were documented on a standardised paper sheet. Technology-related deviations of the recording parameters of the resuscitation simulator were previously calculated. A tolerance of ± 5% was accepted. The check of unconsciousness, the check of breathing, and the shout for help were recorded dichotomously (done/not done). The other parameters were recorded concerning their effectiveness and by stopwatch.

The data were collected with the MS Excel 2003 table calculation programme (Microsoft^®^, Inc. 2003). The statistical analyses were performed by using SPSS for Windows, release 12.0; SPSS, Chicago Ill. Wilcoxon signed rank test, t-test and Fisher's exact test were used where appropriate. Descriptive values of variables are expressed as average, standard deviation and percentages. Power analysis was made by using G-Power™ 3.0.10 for Microsoft Windows XP™ (Microsoft Inc., USA). Wilcoxon signed rank test, t-test and Fisher's exact test were used where appropriate. For data not normally distributed Wilcoxon signed rank test was used (inter group testing; e.g. time data; "No flow time"). Descriptive values of variables are expressed as total, means, median, and percentages. Confidence interval was measured were necessary. All p values of less than 0.05 were considered to indicate statistical significance.

According to the declaration of Helsinki [17] data were made anonymous. An institutional review board approval exists for this study. There was a positive vote of the local ethical commission (an ethical approval was not needed for the execution of this study). Limitations of the cross-over design may be the period (time) effect, the sequence effect, and the treatment effect.

Written consent was obtained after information of the trainees about the investigation at the beginning of the course. All participants were included in the test. Participation in the study and the result of the test had no influence on the certification of the attendance of the course.

The investigation included the following target parameters:

- Checking the state of consciousness of the "patient" (manikin)

- Shout for help, knowledge of the emergency number in Germany

- Check for breathing for at least 10 seconds.

- Effective chest compression

- Effective ventilation

- Correct coordination of the compressions and ventilations.

Besides that the following demographic and further data of the participants were recorded:

1. Age

2. Sex

3. Attendance of previous BLS courses

4. Motivation concerning attendance of the course

5. No-Flow-Time (NFT); the calculated NFT resulted from the definition of the parameters of unconsciousness (15 s) and the 5 × 2 ventilations during the scenario (10 s).

The success parameters which have been previously described followed the requirements defined by ERC in the guidelines 2005 [3]. Every participant had to confirm unconsciousness, to arrange for the emergency call and to perform a cycle of cardiopulmonary resuscitation during a simulated cardiac arrest scenario on a manikin.

Chest compressions proved to be effective if 75% of the compressions were performed with a compression depth of 38–50 mm and the correct hand position. Compressions had to be performed at a rate of 100/min (variation tolerance 10% corresponds to 90–110/min).

The ventilation was considered as effective if it was performed generating a tidal volume of 500–600 ml within one second per ventilation (inspiration and expiration time). By means of the computer recording of the manikin and of the video recording all ventilations performed per participant were retrospectively evaluated. As in the case of chest compression 75% correct ventilations were considered to be effective for the total evaluation. Besides that it was important that compressions and ventilations were performed at a ratio of 30:2.

BLS was defined as effective when all skills were done concerning the 2005 ERC guidelines for BLS [3].

Defined endpoint was the total performance of a whole resuscitation cycle including 5 × 30:2 compressions: ventilations. According to the ERC guidelines 2005 the performance of the cycle was called successful only if it was carried out within a maximum time of 120 seconds.

## Results

Within three months a total of N = 100 participants were included in the study. An average of 10 participants took part in each investigated course (range 8–14 participants).

The demographic data of the participants are reported in Table 1. The study subjects were divided into participants with previous and without previous knowledge concerning BLS. There was an average time interval between this study and the previous courses of two years (range 0.5–8 years). Concerning this time interval, no statistically significant difference could be found.

**Table 1:** Data of the participants

	**Whole Participants ** **N = 100**	**Participants with previous knowledge ** **n = 52**	**Participants without previous knowledge ** **n = 48**
**Sex**	**Number (%)**	**Number (%)**	**Number (%)**
Female	64 (64%)	29 (55.8%)	35 (72.9%)
Male	36 (36%)	23 (44.2%)	13 (27.1%)
			
**Age (years)**	**Number (%)**	**Number (%)**	**Number (%)**
< 20	64 (64%)	31 (59.6%)	33 (68.8%)
21–30	12 (12%)	9 (17.3%)	3 (6.3%)
31–40	7 (7%)	4 (7.7%)	3 (6.3%)
41–50	17 (17%)	8 (15.4%)	9 (18.6%)
			
**Motivation**	**Number (%)**	**Number (%)**	**Number (%)**
driving licence	81 (81%)	41 (78.8%)	40 (83.3%)
Repetition/Interest	19 (19%)	11 (21.2%)	8 (16.7%)
			
**Repetition of the course**	**Number (%)**	**Number (%)**	**Number (%)**
significant	95 (95%)	49 (94.2%)	46 (95.8%)
not significant	5 (5%)	3 (5.8%)	2 (4.2%)

Unconsciousness was confirmed correctly by a total of 94% of the participants; 6% forgot this step. In this respect there was no significant difference between the two groups.

The emergency call was correctly carried out by 65 participants (65%). Comparing the two groups 39 participants of group 1 (75% of this group) and 26 participants of group 2 (54% of this group) performed the emergency call correctly (p = 0.037). Significantly more participants with previous knowledge knew the correct emergency call number and also thought, during the simulation, of communicating early the need of help.

After the inspection the participants had to check ventilation. This check was within 10 seconds effectively carried out by four participants (4%), all of them from the group with previous knowledge. 30% of the participants, also with previous BLS knowledge, performed a check that was not in accordance with the ERC guidelines. 66% of the participants did not check for breathing at all (18 participants with previous knowledge, 48 participants without previous knowledge). In this respect (correct check of ventilation) there was no significant difference between the two groups.

The total time allowed for diagnostics amounted to an average of 14.3 s (median 15.5 s; range 3–40 s; standard deviation 6.5 s). In a total of 14 participants (14%) the time was extended since they performed two initial ventilations, as recommended in the ERC guidelines 2000 [18]. These initial ventilations were carried out only by participants with previous knowledge. Taking this into account, the time until the beginning of the chest compressions amounted to 15.8 s (median 17 s; range 3–40 s; standard deviation 6.4 s).

For performing the CPR cycle, which according to the guidelines of the ERC should not last more than 120 s, the participants needed on average 131.4 s (median 132.5 s; range 89–247 s; standard deviation 30.4 s). Participants with previous knowledge needed 127.1 s (median 130 s; range 97–200 s; standard deviation 23.9 s), while participants without previous knowledge needed significantly more time for the performance of a CPR cycle (average 136.1 s; median 130 s; range 89–247 s, standard deviation 35.6 s).

For the 30 chest compressions participants needed an average of 18.1 s (median 18.2 s; range 11.2–35.4 s; standard deviation 4.5 s; 95%-CI ± 0.3); there was no statistically significant difference between the two groups (**group 1: **average of 17.8 s; median 17.6 s; standard deviation 3.8 s; 95%-CI ± 0.36 **group 2: **average of 18.5 s; median 17.8 s; standard deviation 5.1 s; 95%-CI ± 0.49).

For ventilation the participants needed an average of 8.2 s (median 8.5 s; range 3.8–19 s; standard deviation 2.7 s; 95%-CI ± 0.18) during a cycle including the changeover time between chest compressions and ventilation. Participants with previous knowledge needed less time per ventilation (including the changeover time between chest compressions and ventilations) than participants with no previous knowledge (**group 1: **average of 7,5 s; median 7.2 s; range 3.8–12.8 s; standard deviation 1.8 s; 95%-CI ± 0.17 vs. **group 2: **average of 8.9 s; median 9 s; range 4–19 s; standard deviation 3.2 s; 95%-CI ± 0.31). However, there was no statistically significant difference between both groups.

In the whole group the NFT (time during which no chest compression was performed) amounted to 56.3 s. NFT of participants with previous knowledge was significantly shorter that of participants with no previous knowledge (**group 1: **average of 52.4 s; median 52 s vs. **group 2: **60.7 s; median 59 s; p = 0.042). All times are shown in Table 2 and 3.

**Table 2:** Average (range) time needed for thirty chest compressions, airway management and "No Flow Time" (NFT)

	**Chest compression average (range)**	**Airway management average (range)**	**NFT total average (range)**	**NFT as percent of the whole scenario average (range)**
**Whole participants**	18.1 s (11.2–35.4 s)	8.2 s (3.8–19 s)	56.3 s (29–113 s)	38.5% (20.1–55.4%)
**Participants with previous knowledge**	17.8 s (11.6–32.4)	7.5 s (3.8–12.8 s)	52.4 s (29–86 s)	37.1% (24.3–51.5%)
**Participants without previous knowledge**	18.5 s (11.2–35.4 s)	8.9 s (4–19 s)	60.7 s (33–113 s)	40.1% (20.1–55.4%)
**p value**	ns	ns	**p = 0.042**	**p = 0.039**

**Table 3:** Time needed for cardiopulmonary resuscitation (1 cycle over 120 seconds corresponding to each time 5 × 30 chest compressions and 5 × 2 ventilations [with no consciousness checking])

	**< 120 sec**	**120–130 sec**	**131–140 sec**	**141–150 sec**	**151–160 sec**	**> 160 sec**
**Whole participants**	41 (41%)	13 (13%)	17 (17%)	10 (10%)	6 (6%)	13 (13%)
**Participants with previous knowledge**	23 (44.2%)	7 (13.5%)	11 (21.2%)	5 (9.6%)	2 (3.8%)	4 (7.7%)
**Participants without previous knowledge**	18 (37.5%)	6 (12.5%)	6 (12.5%)	5 (10.5%)	4 (8.3%)	9 (18.7%)
**p value**	ns	ns	ns	ns	ns	ns

More than 75% effective chest compressions with correct hand position, pressure depth and frequency were performed by a total of 30 participants, in accordance with the 2005 ERC guidelines [3]. 22 participants with previous knowledge (44% of this group) performed the chest compressions correctly, significantly more than in the group of participants with no previous knowledge (8 participants, 17%; p = 0.0058). In 25% out of the latter group the hand position deviated essentially from the current guidelines, so that injuries of other organ structures might be probable.

Concerning ventilation it was expected that participants with previous knowledge would perform more correct ventilations than participants with no previous knowledge (**group 1: **44%, **group 2: **21%; p = 0.0062). The results of chest compression and ventilation are reported in Table 4.

**Table 4:** Effectiveness of diagnostics, airway management and chest compression

**Measure**	**Participants with previous knowledge ** **(n = 52)**	**Participants without previous knowledge ** **(n = 48)**	**p value**	**whole participants ** **(n = 100)**
	**effective**	**effective**		**effective**
**Reaction**	50 (96.2%)	44 (91.7%)	ns	94 (94%)
**Emergency call**	39 (75%)	26 (54.2%)	**p = 0.037**	65 (65%)
**Breath control**	4 (7.7%)	0 (0%)	ns	4 (4%)
**Chest compression**	22 (42.3%)	8 (16.7%)	**p = 0.0058**	30 (30%)
**Airway management**	23 (44.2%)	10 (20.8%)	**p = 0.0062**	33 (33%)

In total, BLS was carried out significantly better by participants with relevant previous knowledge, although there was no significant difference between the groups concerning the diagnostic steps of checking for responsiveness and breathing.

Therefore an effective cardiopulmonary resuscitation according to the ERC guidelines 2005 [3] was performed by a total of 22 participants (22%; **group 1: **n = 17, 33%; **group 2: **n = 5, 10%; p = 0.0038; Table 5).

**Table 5:** BLS/Resuscitation effectiveness

	**effective**	**Non effective**
**Whole participants (n = 100)**	22 (22%)	78 (78%)
**Participants with previous knowledge (n = 52)**	17 (32.7%)	35 (67.3%)
**Participants without previous knowledge (n = 48)**	5 (10.4%)	43 (89.6%)
**P value**	**P = 0.0038**	ns

## Discussion

**Figure 1:**
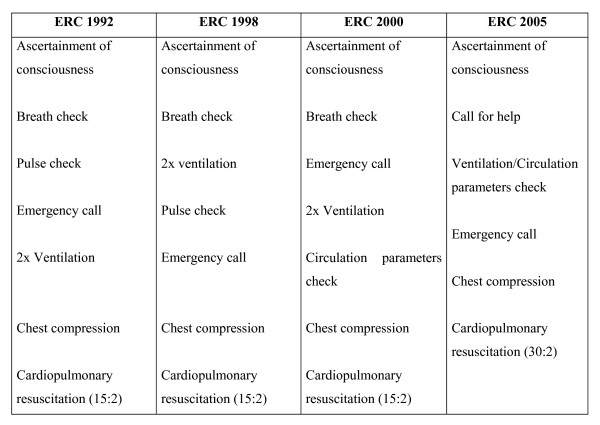
**Differences of the guidelines** [[3,18,20],** and**[21]].

Four versions of BLS guidelines for adults have been published by the ERC since 1992 [[3,18,19], and [20]]. The relevant changes in the guidelines are shown in Figure 1.

A modification in the last years concerned the extent of the respiratory volume and the duration of single ventilation [[3,18,19], and [20]] to obtain an interruption of chest compression as short as possible. In our investigation we could find that the times for every ventilation cycle (including the changeover from chest compression to ventilation) lasted on average 8.2 s, adding up to more than 40 s for one resuscitation cycle with 5 × 2 ventilations. This time represents a large part of the total NFT. A significant difference between the two groups as far as the ventilation time is concerned could not be found. With regard to some studies that reviewed critically the performance of ventilation by lay people, a revision of BLS, especially concerning ventilation, should be taken into consideration [21-24].

Concerning these many changes group 2 should have performed BLS better than group 1, because they did not have to replace their existing knowledge. However, in the present investigation, we were not able to show such a result.

In the present study only 22% of all the participants managed to perform an effective resuscitation according to the current ERC guidelines for BLS [3]. Therefore the major result of the present study was that overall performance of resuscitation was poor. Similar results could be shown in earlier manikin studies [9]. If participants with previous knowledge in BLS are compared with participants without, significant differences appear in the effectiveness of the performance of BLS on the manikin (p < 0.01) (**group 1: **32.7% vs. **group 2: **10.4%; p < 0.01). Hence, the question of obligatory refresher courses should at least be discussed [[8,9], and [25]]. However, even a modification of the course structures seems to be worth considering, as even only 30% of the participants with previous knowledge were able to perform all BLS steps effectively on the manikin. Our finding that group 1 took less time to complete the CPR cycle, but both groups' times failed to meet the ERC standard has to be mentioned. Therefore most participants were not able to perform the CPR cycle in according to the ERC guidelines.

### Importance of BLS

The most frequent causes for the necessity of bystander resuscitation are of cardiac origin [[3,26], and [27]], with sudden cardiac death being the most important indication for resuscitation [26,27]. Already in the 1950's it was demonstrated that chest compressions only could generate a ventilation flow and have a positive effect on outcome – a finding that justifies the early beginning of chest compression [28,29]. Also in the current ERC guidelines the importance of chest compressions is stressed [3]. Assuming that mouth-to-mouth ventilation might have no positive effect on outcome [[21,23], and [24]], chest compressions are the factor influencing the success of bystander resuscitation. For this reason chest compressions should be performed as early and as effectively as possible, since by correct chest compression ventricular fibrillation as well as a minimal circulation could be maintained [30,31]. In our study only 30% of the participants performed effective chest compressions on the manikin. This shows that improvements in training are urgently needed.

A compression depth of 38–50 mm at a rate of 100 per minute is necessary for effective chest compression [3]. The pressure depth plays an important role concerning injuries of chest and abdominal organs [31]. In a previous investigation injuries of the chest (70%), of the heart and of the lungs (30%) were found post mortem in 97% of victims resuscitated by physicians [32]. In our study 25% of the participants performed potentially dangerous chest compressions with wrong hand position and a clearly too great pressure depth. Again, improvements are urgently required.

Several clinical and experimental surveys have led to various recommendations as to the importance of ventilation during BLS [[3,22-24], and [33-36]]. This is still reflected in the current ERC BLS guidelines [3]. In our investigation most of the participants did not succeed in performing effective ventilation in an acceptable time interval.

The current guidelines state that within a CPR cycle of two minutes ten breaths should be given. In our study participants needed on average 8.2 s for two ventilations (including the changeover time between chest compression and ventilation). Further investigations in this field to evaluate the duration of ventilation and its effectiveness during BLS should therefore be carried out and critically discussed.

Reith et al. investigated in various groups of lay people their ability to assess correctly a respiratory arrest [37]. This was the first scientific study about the effectiveness of checking for breath. In all investigated groups the quality of the check was considered insufficient so that a better training with regular repetition seemed indispensable [37]. This has been confirmed also in our investigation (96% of the investigated participants checked breathing unsatisfactorily), although it has to be critically mentioned that this has been a simulation study and the participants were aware that the "patient" (the manikin) had a respiratory arrest. In comparison with another manikin study the results could however be confirmed since in this earlier work it was shown that the participants could assess a respiratory arrest only unsatisfactorily [9]. Thus, a correct performance of checking for breathing according to the current ERC guidelines [3] seems to be a difficult procedure for a lot of lay people.

In case of a loss of vital functions CPR by lay people includes their restoration and maintenance up to the arrival of professional help. Already four to five minutes after occurrence of cardiac arrest irreversible cerebral damage may appear. Therefore resuscitation measures must begin as soon as possible, especially since average response times of the professional EMS system in Germany clearly exceed five minutes so that lay people have to bridge the gap [[3,26], and [30]]. In according to this the differences between group 1 and group 2 were far less interesting than the differences between the groups overall numbers, and the desirable level of performance. To verifying the study results more clinical investigations are necessary.

### Weaknesses of the study

Our study has a number of inherent limitations. The investigation does not have sufficient power to perform subgroup analysis of each individual course.

An investigation on a manikin can only partially be transferred to a pre-hospital situation. Therefore it is possible to have false-positive and false-negative evaluations on the manikin in comparison with reality. In studies on patient outcomes for example the effectiveness of chest compression is assessed by a palpable peripheral pulse and the effectiveness of ventilation by a raising and falling of the thorax [39,40] [41]. However, a model of an effectiveness test in this form is not comparatively feasible, so it is necessary to make reference to validated and recommended parameters (for example pressure depth 38–50 mm, tidal volume 500–600 ml).

The authors also have to separate the concepts of a clinically significant versus a statistically significant difference in the parameters measured (e.g. time parameters concerning NFT). P values can be statistically significant, but the clinical significance of the difference may be negligible. Therefore, clinical investigations are necessary.

Furthermore participants in manikin exercises are aware that the "patient"does not breathe and therefore the check may possibly be carried out more ineffectively than in reality. Besides that, stressors (psycho-social, moral and ethical) during a real cardiac arrest situation may in both directions influence the way of performing BLS. For this reason the obtained results cannot directly be transferred to a real cardiac arrest situation including outcome. However, a manikin study can indicate weak points in bystander resuscitation and in the respective first aid courses, the improvement of which should be an important aim for the future.

Participants who had the impetus to present for an additional training may have been more motivated (and thus possibly more likely to perform well regardless of course repetition).

In some respects, such as the time since the previous BLS course was taken, the study n allows no robust comparison. The study endpoints have been defined in accordance with the ERC guidelines of 2005. This removes an important source of subjectivity in the study methodology.

## Conclusion

Our study has shown that most of the investigated participants even immediately after the "LSM course" were unable to transfer successfully what they had just learned in a BLS testing scenario. Participants with previous knowledge were significantly better in performing BLS than those without previous knowledge. For this reason the repetition of specific course contents seems to be essential for improvement of the resuscitation skills. In addition further simplifications of BLS (for example concentration on chest compressions) might increase the training effects as well as the effectiveness of bystander resuscitation. This should be investigated in further clinical studies including patient outcome.

## Competing interests

The authors declare that they have no competing interests.

## Authors' contributions

CW has made substantial contributions to conception and design, analysis and interpretation of data. HW has made substantial contributions to acquisition of data. JB has been involved in drafting the manuscript or revising it critically for important intellectual content. BMG has made substantial contributions to conception of the investigation.

## Pre-publication history

The pre-publication history for this paper can be accessed here:


